# Potential factors involved in the causation of rhabdomyolysis following status asthmaticus

**DOI:** 10.1186/s13223-016-0149-6

**Published:** 2016-08-22

**Authors:** Hongmei Qiao, Huanji Cheng, Li Liu, Jianing Yin

**Affiliations:** Department of Pediatrics, The First Hospital of Jilin University, Xinmin street 71#, Changchun, 130021 Jilin Province People’s Republic of China

**Keywords:** Rhabdomyolysis, Status asthmaticus, Risk factors, Respiratory muscle exertion, Medication, Mechanical ventilation

## Abstract

Rhabdomyolysis is a rare but potentially fatal complication of status asthmaticus. Since the first case was reported in 1978, only a few dozen cases have been described till date. We performed a literature review with the aim to characterize the pathophysiological basis of the occurrence of rhabdomyolysis in patients with status asthmaticus. Excessive exertion of respiratory muscles, hypoxia and acidosis, electrolyte imbalance, infections, some drugs used for asthma control, use of mechanical ventilation, prolonged cardiopulmonary resuscitation, higher age of the patient and some underlying diseases or genetic factors appear to be involved in its causation. In patients with status asthmaticus, it is important to pay more attention to these factors and to closely monitor creatine kinase levels in blood so as to ensure early detection of rhabdomyolysis.

## Background

Rhabdomyolysis, meaning destruction or disintegration of striated muscles, is characterized by muscle breakdown and necrosis that results in the leakage of intracellular muscle constituents into the circulation and extracellular fluids [1]. The condition may range from an asymptomatic illness with elevation in the creatine kinase (CK) to a life threatening condition associated with extreme elevation in CK levels, electrolyte imbalance, acute renal failure and disseminated intravascular coagulation. Rhabdomyolysis may occur in various congenital and acquired medical conditions, but rarely occurs as a complication of status asthmaticus.

The first reported case of rhabdomyolysis secondary to status asthmaticus was described by Chugh et al. in 1978 [2]. Since then, only tens of cases of rhabdomyolysis following status asthmaticus have been reported in the last four decades. Most of these patients were adults; only six papers involved children and teenagers aged between 4 and 18 years [3–8].

In this paper, we sought to characterize the pathophysiological basis of the occurrence of rhabdomyolysis in patients with status asthmaticus. A literature search for relevant publications was performed; clinical investigations, reviews and case reports were also included. A discussion on the factors that may precipitate the development of this extremely rare complication of status asthmaticus is presented (Table 1).

**Table 1:** Potential factors involved in the causation of rhabdomyolysis secondary to status asthmaticus

Physiological factors	Exertion of the respiratory muscles Hypoxia and acidosis Electrolyte imbalances
Infections	Bacteria: streptococcus, pneumococcus, legionnaire, etc. Virus: influenza, etc. *Mycoplasma pneumoniae*
Medications	Glucocorticoids β_2_-Adrenergic agonists Theophylline Propofol Neuromuscular blocking agents Antibiotics (fluoroquinolone, clarithromycin, amphotericin B, etc.)
Relevant therapy	Resuscitation Mechanical ventilation
Patient factors	Underlying diseases Age Genetic heterogeneity

### Physiological factors

#### Exertion of respiratory muscles

Several factors may be involved in the pathogenesis of rhabdomyolysis, among which muscle exertion is the most common cause in both adults and children [9]. The underlying mechanism of rhabdomyolysis following severe exertion appears to involve a combination of mechanical and thermal muscle injury and ATP depletion [10]. Vigorous and repeated contractions of the respiratory and accessory muscles coupled with hypoxia appear to be the underlying mechanism of muscle damage and cell necrosis in patients with status asthmaticus [2, 4, 6, 7]. Risk factors for exertional rhabdomyolysis include, hypokalemia, sickle-cell trait [11], extreme heat and humidity, or pre-exertion fatigue. These conditions should be watched out for and avoided in patients with severe asthma attack. In addition, repeated coughing during the attack can also aggravate the severity of muscular fatigue [2].

#### Hypoxia and acidosis

Generalized hypoxemia due to respiratory insufficiency secondary to status asthmaticus can lead to muscular ischemia and injury, which is regarded as another cause of rhabdomyolysis, due to its association with insufficient ATP production and sarcolemmal dysfunction [12]. Hypercapnia was a common finding in most cases of rhabdomyolysis associated with status asthmaticus. The occurrence of metabolic acidosis may identify patients at greater risk for rhabdomyolysis [7]. Some authors even point out that the combination of respiratory and metabolic acidosis may increase corticosteroid receptors and aggravate the risk of corticosteroid-induced muscle injury [3].

In most of the previous reported cases, vigorous exertion of respiratory muscles, generalized muscle hypoxia, and metabolic and respiratory acidosis were often found together and considered to be the main factors leading to rhabdomyolysis [2, 4, 7, 13, 14].

#### Electrolyte imbalance

Electrolyte imbalance is a common complication in various diseases, and status asthmaticus is no exception. Hypokalemia, hypophosphatemia, hyponatremia and even rapid correction of hyponatremia [15–17], have been reported to induce rhabdomyolysis. Among these, hypokalemia is considered to be the most notorious one. Hypokalemia is known to depolarize the muscle membrane, inhibit the production and storage of glycogen in myocytes [1], and even limit vasodilatation in the muscle microvasculature during exercise [18]. These appear to be the underlying mechanisms of the association between hypokalemia and exertional rhabdomyolysis. It is likely that electrolyte imbalance may trigger rhabdomyolysis during acute exacerbation of asthma; avoidance of hypokalemia in these patients is of great importance.

### Infections

Asthma exacerbation can be triggered by various infections, especially that by viruses and mycoplasma pneumoniae. However, infection can also be a precipitating factor of rhabdomyolysis due to direct tissue damage, effect of toxins [19], inflammatory mediators [20], or associated fever, acidosis and dehydration [21, 22]. Pathogens reportedly associated with rhabdomyolysis include streptococcus [23], pneumococcus [21], legionella [24], influenza [25] (including recent pandemic of H1N1 subtype [26]), and mycoplasma pneumoniae [27]. Thus, we suggest the detection of multi-species pathogens in these patients.

### Medications

#### Glucocorticoids

Glucocorticoids are one of the most important groups of medications used for asthma control. It is also well known to cause muscle injury, especially with prolonged therapy at high doses. Usually, glucocorticoid associated myopathy is characterized by a slowly-progressive, predominantly proximal muscle weakness and atrophy. Rhabdomyolysis occurring as a complication of glucocorticoid therapy has rarely been reported [3, 28]. The underlying mechanism of glucocorticoid-induced myotoxicity is not completely understood. In addition to suppressed protein synthesis and enhanced proteolysis of myofibrillar proteins, a recent review focused on one potential mechanism of glucocorticoid-induced apoptosis in skeletal muscle, i.e., through mitochondrial-mediated and Fas-mediated apoptosis, leading to activation of proteasome in skeletal muscle, suppression of IGF-1 signaling and generation of ceramide [29].

The development and the severity of glucocorticoid-induced muscular injury shows much inter-individual variability and appears to depend on the treatment regimen, duration and dosage (repetitive burst treatment effects are considered worse than continuous treatment with the same dose) [30]. During short-term treatment with massive doses of glucocorticoids as frequently used in status asthmaticus, acute severe muscle injury may develop and is characterized by generalized fiber necrosis and rhabdomyolysis [30].

Administration of neuromuscular blocking agents (NMBAs) in patients with severe asthma attack that requires mechanical ventilation may lead to myopathy. The combined effects of the NMBAs and glucocorticoids, rather than either type of drug alone, could lead to myopathic outcomes, which may range from asymptomatic CK elevation to myopathy with weakness [31–33] and even rhabdomyolysis [3]. Electromyographic findings suggest that the muscular denervated state due to the absence of cholinergic stimulation caused by NMBAs could potentiate the effect of glucocorticoids by inducing an increase in the cytoplasmic corticosteroid receptors [34, 35] and promote the depletion of myosin [36].

Inhalational corticosteroid therapy is much safer than systemic administration; there is no evidence of rhabdomyolysis directly induced by inhaled glucocorticoids.

#### β_2_-Adrenergic agonists

β_2_-Adrenergic agonists, one of the main treatment modalities for asthma, are commonly used during acute exacerbations. β_2_-Adrenergic agonists have been reported to induce muscle cramps and weakness, which is associated with varying degrees of CK elevation [37, 38]. However, rhabdomyolysis secondary to β_2_-adrenergic agonist therapy is indeed rare. Only in one case, rhabdomyolysis and acute renal failure after terbutaline overdose was reported, and the authors proposed that the hyperkinetic effects of intense β_2_-receptor stimulation may have induced rhabdomyolysis [39]. The definitive mechanism is not clear, but tremor is a common side effect of β_2_-adrenergic agonist and hypokalemia can occur due to increased Na^+^/K^+^ ATPase activity in cases of β_2_-adrenergic agonist poisoning [40]. There are no records of rhabdomyolysis induced by nebulized β_2_-adrenergic agonists.

#### Theophylline

Rhabdomyolysis is a rare complication of theophylline intoxication. Nearly all cases of theophylline related rhabdomyolysis in previous case reports were associated with large doses [41–43]. Therapeutic dose of theophylline is unlikely to cause muscle injury. An 81-year-old asthmatic male who developed rhabdomyolysis after intravenous administration of 250 mg aminophylline appears to be an exception [44]. Development of rhabdomyolysis after theophylline occurred, presumably, at least in part, due to other complications of theophylline toxicity, such as profound hypokalemia, seizures or hypotension [45–47]. Therefore, in status asthmaticus patients on theophylline therapy, it is better to monitor serum theophylline levels and other features of theophylline toxicity such as seizures and deranged potassium homeostasis.

Theophylline is often administered with drugs that may elevate the serum CK level (CK-elevating drugs) such as β_2_-adrenergic agonists and corticosteroids in status asthmaticus patients. A study by Iwano et al. [48] suggested that combined treatment of theophylline and CK-elevating drugs may induce a synergistic increase in the serum CK levels. We speculate that it is the synergistic effect of several factors that leads to rhabdomyolysis during status asthmaticus; this may also explain why rhabdomyolysis is not commonly described in other severe respiratory diseases such as exacerbation of COPD, in which exertion of the respiratory muscles, hypoxia and acidosis are common features.

#### Sedatives

Propofol is a popular anesthetic and sedative agent due to its rapid onset of action and short half-life, decrease in cerebral oxygen consumption, and the associated reduction in intracranial pressure [49]. Sedation with propofol is often required to facilitate ventilator synchrony during mechanical ventilation in severe asthmatic patients. However, rhabdomyolysis, occurring as part of propofol-related infusion syndrome (PRIS), has been sporadically reported in patients on prolonged propofol therapy in intensive care units (ICU) [50].

Various pathophysiological mechanisms of occurrence of rhabdomyolysis in PRIS have been postulated. Propofol impairs the mitochondrial respiratory chain and production of ATP, and further increases triglyceride load and disturbs the fatty acid oxidation. The deranged lipid and lactate metabolism in cardiac and skeletal muscles, along with impaired ATP synthesis, contributes to myocytolysis, leading on to myocardial failure, arrhythmia, and rhabdomyolysis [51–54]. Pathological findings of cytolysis of skeletal and cardiac muscle cells in propofol infusion syndrome have been documented [55]. Other triggering factors include low carbohydrate supply, excess catecholamines and corticosteroid therapy in critically ill patients, which can cause myocyte injury, lipolysis, and increase circulating free fatty acids [52, 56].

On the whole, a lower propofol dose for a shorter duration with adequate carbohydrate intake is preferable. For adults, no more than 4 mg/kg of propofol per hour for a maximum of 48 h, is recommended [52]. Children are more prone to the development of PRIS due to low glycogen storage and high dependence on fat metabolism [57].

In 2001, the US Food and Drug Administration placed a warning against use of propofol for long-term sedation in pediatric patients. The maximum permissible dose of propofol for sedation in the pediatric ICU is <5 mg/kg per hour, under strict monitoring of blood lactate, myoglobin and CK levels [58]. For patients suffering from status asthmaticus who need mechanical ventilation, propofol seems to be a high-risk factor for rhabdomyolysis. It is important for clinicians to keep this undesirable complication in mind and to monitor for early warning signs, because prompt cessation of propofol infusion can reverse the impending consequences at an early stage [51]. Alternative drugs include lorazepam and midazolam [49].

#### Antibiotics

Exacerbation of asthma is sometimes induced by lung infection, and antibiotics might be used in this setting. Administration of antibiotics such as fluoroquinolone [59], levofloxacin [60], clarithromycin [61], linezolid [62] and amphotericin B [63] is reportedly associated with rhabdomyolysis. Monitoring of serum CK levels is recommended alongside the use of any of these antibiotics in asthmatic patients.

Cytochrome P450 (CYP) 3A4 is known to oxidize a wide range of drugs, and effect drug metabolism. Clinically important CYP3A4 inhibitors include itraconazole, ketoconazole, clarithromycin, erythromycin, etc. Rhabdomyolysis is known to be a serious consequence of the co-administration of statins and CYP3A4 inhibitors. Concomitant use of clarithromycin with theophylline was reported to induce rhabdomyolysis in an asthmatic patient, by enhancing theophylline toxicity through interfering with its metabolism [64]. Drug interactions may be most apparent when patients are stabilized on an affected drug and the CYP3A4 inhibitor is then added to the regimen [65]. For instance, a patient with corticosteroid-dependent asthma and allergic bronchopulmonary aspergillosis developed rhabdomyolysis after itraconazole supplementation to her long-time corticosteroid (deflazacort) treatment [66].

The drugs mentioned above are commonly used during asthma exacerbation; interaction between these drugs may potentially induce rhabdomyolysis during treatment of status asthmaticus. Nonetheless, status asthmaticus should be treated aggressively at an early stage in these patients to prevent respiratory muscle fatigue, muscle hypoxia, and to minimize the risk of postexertional rhabdomyolysis [3, 4, 7].

### Other therapeutic interventions

#### Resuscitation

A case of rhabdomyolysis that occurred after successful resuscitation from near-fatal asthma is on record [14]. According to the authors, the most likely etiology of rhabdomyolysis in this patient was vigorous and repeated contractions of respiratory muscles with muscular hypoxia; however, the association of cardiopulmonary resuscitation (CPR) and rhabdomyolysis should be further assessed. Based on the two cases of post-resuscitational rhabdomyolysis, the possible mechanisms may have been administration of excessive amounts of epinephrine, massive cardioversion and prolonged CPR [67, 68].

#### Mechanical ventilation

According to previous case reports, most cases of rhabdomyolysis following status asthmaticus had a history of endotracheal intubation and mechanical ventilation [3, 4, 6, 7, 14]. In a retrospective review, rhabdomyolysis, as a rare complication of asthma, showed a much higher frequency (incidence: 8 %) in intubated asthmatic children than in non-intubated ones [5].

The available reports indicate that conditions associated with mechanical ventilation may play a contributory role in the development of rhabdomyolysis. Patients who need mechanical ventilation have more serious hypoxia and/or acidosis which is likely to contribute to the development of rhabdomyolysis. Secondly, propofol and sometimes NMBAs are needed to facilitate mechanical ventilation. Concomitant use of these two alongside massive doses of glucocorticoids is likely to enhance the risk of muscle damage. Besides, prolonged immobilization in patients under mechanical ventilation can induce tissue compression and muscle ischemia, which may also increase the risk of rhabdomyolysis [69, 70]. Additionally, strategy of permissive hypercapnia is widely recommended in patients with status asthmaticus to minimize hyperinflation and ventilator-induced lung injury. However hypercapnia may be a risk factor for rhabdomyolysis [3].

What should also be highlighted is the relationship between the development of rhabdomyolysis and barotrauma or ventilator-induced myotoxic cytokines. However, there are no correlational studies, and it may be a new direction to explore.

### Patient factors

#### Underlying diseases

Repeated occurrence of rhabdomyolysis after asthmatic attacks in a patient with McArdle disease (myophosphorylase deficiency) is on record [71]. In another case report, a soldier developed severe rhabdomyolysis after a mild acute asthma exacerbation. Further work-up revealed an underlying deficiency of type II carnitine palmitoyltransferase [72]. Both these underlying diseases belong to metabolic syndromes that are more sensitive to oxygen insufficiency and manifest as exercise intolerance. It suggests that muscle damage would happen more easily during asthma exacerbation given the underlying diseases.

Besides these metabolic syndromes, some endocrine disorders (such as thyroid storm [73], diabetic ketoacidosis [74]) and autoimmune diseases (such as dermatomyositis [75]) have all been shown to promote rhabdomyolysis. Thus, the occurrence of rhabdomyolysis in the context of asthma exacerbations should warrant a further work-up for underlying diseases.

#### Age

In a retrospective review by Mehta et al. [6], children who developed acute rhabdomyolysis tended to be older than those who did not develop rhabdomyolysis (median age 15 vs. 5 years, respectively). They inferred that the relatively lower muscle mass in the younger children may have prevented the development of exertional rhabdomyolysis. Most previous reports pertain to adults or teenagers. Only one case report pertained to a 4-year-old boy, who is the youngest patient with this medical condition to the best of our knowledge [8].

#### Genetic heterogeneity

Most patients experience only one episode of rhabdomyolysis. However, in case of recurrent rhabdomyolysis, a history of exercise intolerance or a positive family history for neuromuscular disorders should be actively sought. Further investigations are also needed to identify any underlying genetic disorder [76].

The influence of genetic variations on statin-induced rhabdomyolysis has been investigated in a candidate gene association study and whole-genome sequencing [77]. We could not identify any studies that directly investigated the effect of genetic heterogeneity on rhabdomyolysis associated with status asthmaticus. However, several gene-specific single nucleotide polymorphisms (SNPs) are reportedly associated with severe exercise-induced muscle damage. These include CKMM Ncol, ACTN3 R577X, and MYLK C37885A [78, 79].

This knowledge base may be useful for clinicians in identifying status asthmaticus individuals who are more susceptible to rhabdomyolysis. This is surely a new field for further exploration.

## Conclusion

Rhabdomyolysis, a rare but potentially fatal complication of status asthmaticus, calls for more attention. Current literature suggests that severity of hypoxia, acidosis and exertion of respiratory muscles, infections and/or electrolyte imbalance, the use of mechanical ventilation, prolonged CPR, large dosages of corticosteroids or theophylline in combination with NMBAs or CYP3A4 (such as macrolides), sedation with propofol, older age, and underlying myopathy may be the risk factors for rhabdomyolysis in patients with status asthmaticus. Research into genetic susceptibility to rhabdomyolysis is a new field for investigation. Clinicians should pay more attention to the clinical signs (muscle pain, muscle edema, dark-colored urine) and monitor CK levels for early detection of this rare complication. Once rhabdomyolysis occurs, it is important to prevent further muscle breakdown by adequate oxygenation and energy intake, correction of acidosis and electrolyte imbalance, change in sedative agent, etc. Early aggressive hydration may avoid deterioration and prevent irreversible damage.

## Abbreviations

CK: creatine kinase
CPR: cardiopulmonary resuscitation
CYP: cytochrome P450
ICU: intensive care unit
NMBAs: neuromuscular blocking agents
PRIS: propofol-related infusion syndrome
SNPs: single nucleotide polymorphisms
